# Predicting cardiovascular risk with hybrid ensemble learning and explainable AI

**DOI:** 10.1038/s41598-025-01650-7

**Published:** 2025-05-23

**Authors:** Pooja Shah, Madhu Shukla, Neel H. Dholakia, Himanshu Gupta

**Affiliations:** 1 Department of Computer Science and Engineering, Pandit Deendayal Energy University, Knowledge Corridor, Raisan Village, Gandhinagar, Gujarat 382007 India; 2 Department of CSE - AI, ML & DS, Marwadi University, Rajkot, Gujarat 360003 India; 3 Department of Instrumentation and Control Engineering, Manipal Institute of Technology, Manipal Academy of Higher Education, Manipal, Karnataka 576104 India

**Keywords:** Cardiovascular risk prediction, Hybrid ensemble learning, Explainable AI, SHAP Analysis, Multidimensional feature analysis, Cardiovascular diseases, Computer science

## Abstract

Cardiovascular diseases (CVDs) are still one of the leading causes of death globally, underscoring the importance of early and right risk prediction for effective preventive measures and therapeutic approaches. This study proposes an innovative hybrid ensemble learning framework that combines state-of-the-art machine learning models and explainable AI approaches to risk prediction for cardiovascular disease. Using a range of publicly accessible datasets, the suggested structure incorporates Gradient Boosting, CatBoost, and Neural Networks using a stacked ensemble architecture, resulting in more robust predictive performance than the constituent models. This is particularly interesting when visualised through techniques such as SHAP values, t-SNE and PCA projections which allows the study to explore the multidimensional aspects of the relationships between key risk factors including systolic/diastolic blood pressure, BMI, cholesterol-glucose ratio, alongside various lifestyle parameters. They build further on model interpretability through explainable AI methods so that clinicians can observe the involvement of each feature in generating the predictions. The hybrid model demonstrated strong predictive performance with an AUC-ROC score of 0.82, and confusion matrices showing a well-balanced classification of both positive and negative cases - achieving Precision: 81%, Recall: 83%, and F1-Score: 82% on the test dataset. The results highlight the potential of ensemble learning for addressing complex medical prediction problems and the need for models to be interpretable to ensure the trustworthiness of AI systems in healthcare settings. These findings provide an exciting opportunity toward better models of CVD risk prediction, potentially providing healthcare stakeholders with interpretable means to target treatments.

## Introduction

Cardiovascular diseases (CVDs) are a major public health challenge as they are the leading cause of death worldwide^1^. Predicting it with effective intervention early is needed to reduce risk and improve patient outcomes. Through data learning and extension of extensive data, advanced machine-learning models have emerged as a breakthrough method for providing insights into the patterns within heterogeneous^2^ and high-dimensional data in medical science research. Conventional approaches for predicting cardiovascular risk typically make linear assumptions and involve pre-defined relationships between variables, potentially neglecting complex interactions among features. State-of-the-art ensemble learning methods have recently shown to achieve a better predictive performance by taking advantage of heterogeneous different models^3,4^. But the inability of these models to be interpretable has made their use in clinical settings more difficult, where transparency and explainability are key. Here, a hybrid ensemble learning framework for predicting cardiovascular risk by combining the prediction strengths of nearly all state-of-the-art algorithms including LightGBM, XGBoost, CatBoost and neural networks is proposed. A design of the proposed model is used in which the multidimensionality of the problem is captured: multiple engineered features and clustering-based approaches, while the stability of the model for XAI (SHAP i.e., SHapley Additive exPlanations) is explained. It is highlighted that the efficacy of this hybrid approach^5,6^ through experimental comparisons, suggesting that not only do these models provide better predictive power, but also do so in a way that can be interpreted to uncover risk factors of cardiovascular disease^7^. Applying visualization techniques including PCA, t-SNE, and SHAP-based plots, continued to provide insights into underlying relationships in the data, moving from model performance to clinically interpretable output. The knowledge gained from this research will facilitate the development of a transparent and explanation-based framework that meets the demands of clinical decision-making, leading to better-informed and actionable interventions in cardiovascular healthcare.

The main reason behind this study is to enhance cardiovascular disease (CVD) prediction and it is undoubtedly not a trivial one, since the thorough process is a task that remains important when medical data is heterogeneous and high-dimensional. Timely and accurate identification of individuals at risk for CVD could reduce the risk of all-cause mortality and lead to improved outcomes in both prevention and treatment, but traditional models often fail to account for complex, non-linear relationships between features. This work seeks to achieve these two complementary goals by combining state-of-the-art ensemble-learning techniques with Explainable AI (XAI) methods, providing improved predictive performance as well as interpretability-critical to gaining clinical adoption. The capability to measure how various risk factors contribute to a model’s prediction is critical to developing trust and ensuring that AI-driven tools are clinically actionable.

In cardiovascular disease prediction scenario, for example, XAI can help healthcare professionals to understand what each risk factor (BMI, blood pressure, cholesterol levels, etc.) means for a model’s outcome. Transparent mechanisms with techniques such as SHAP (SHapley Additive exPlanations) and visual techniques like PCA and t-SNE enable interpretability of the model’s reasoning process. Such actions are important for gaining trust, establishing the validity of predictions with respect to clinical intelligence, and enabling responsible adoption of AI in sensitive healthcare settings^8^. This explains role of Explainable AI (XAI) in bridging the gap between complex machine learning models and clinical practice.

This study makes five key contributions as follows:

1. In this work, a cardiovascular risk prediction model based on a hybrid ensemble learning framework is proposed that includes Gradient Boosting, CatBoost, LightGBM, SVM, and Neural Network algorithms with XGBoost as a meta-learner.
2. Tackling problems of data imbalance using SMOTE and undersampling techniques to ensure fair training and robust model performance.
3. Incorporated Explainable AI methods (SHAP values, PCA and t-SNE) to interpret the prediction model to allow for clinicians to examine the effects of the individual risk factors.
4. Conducted extensive evaluation and comparison on several datasets and models to show the efficacy and generalizability of the approach.
5. The hybrid model reaches an impressive AUC-ROC of 0.82, showing clinical relevance and possible real world implementation.

Using Explainable AI techniques, such as SHAP values and high-dimensional visualizations such as PCA and t-SNE, the study explains the relationship between the main risk factors and the predictions of models. Over a variety of cardiovascular risk profiles, the proposed approach is thoroughly assessed against numerous datasets so as to guarantee the robustness and generalizability. Also the work addresses class imbalance challenges to make predictions more reliable, even with imbalanced datasets, using sophisticated preprocessing techniques such as SMOTE. The rest of the manuscript is structured as follows: Sects. 2 and 3 presents the motivation and related works on cardiovascular risk prediction. The fourth section goes deeper into the methodology, including data preprocessing, feature engineering, and model architecture and in sect. 5 provides experimental results, including performance evaluation, and visual analysis of explainable AI techniques. Section 6 provides the study’s implications, limitations, and future research directions followed by references and author contribution statement.

**Table 1:** List of Abbreviations.

Abbreviation	Meaning
CVD	Cardiovascular disease
AI	Artificial intelligence
XAI	Explainable artificial intelligence
SHAP	SHapley additive exPlanations
PCA	Principal component analysis
t-SNE	t-distributed stochastic neighbor embedding
SMOTE	Synthetic minority over-sampling technique
AUC-ROC	Area under the receiver operating characteristic curve
SVM	Support vector machine
XGBoost	Extreme gradient boosting
LightGBM	Light gradient boosting machine
CatBoost	Categorical boosting
BMI	Body mass index
ROC	Receiver operating characteristic
TP	True positive
TN	True negative
FP	False positive
FN	False negative

Table 1 provides a comprehensive list of abbreviations used throughout this study. It covers frequently used terms in cardiovascular disease prediction, machine learning models, and Explainable AI (XAI) methods, relieving any confusion with worldings used in this manuscript.

## Motivation

Cardiovascular diseases (CVDs) rank as one of the leading causes of death worldwide, resulting in 17.9 million deaths annually and representing 32% of all global deaths exerting a considerable financial impact on the healthcare sector^9^. While there is much progress made in medical research, it is still difficult to achieve early detection and prevention of CVDs because of the multifactorial nature of the disease and the complexity of the data.

One of the main drawbacks of conventional risk prediction models is the linear assumptions, leading them to be unable to account for the nonlinear and multidimensional relationships naturally existing in medical data. Recent research has indicated that some ensemble machine learning techniques are more accurate than classical models. Because of their non-interpretable nature (“black-box problem”), their use in clinics (where transparency is essential) remains limited. Explainable AI (XAI) serves as a promising solution in this regard by rendering the complex model predictions interpretable and actionable to healthcare professionals. However, few works combine high-performing ensemble models with interpretable XAI frameworks in a clinically robust and scalable fashion.

This is the motivation behind the research - developing an hybrid ensemble learning model that incorporates the best-in-class ML classifiers along with explainability tools like SHAP, PCA, t-SNE, specifically for accurate and interpretable cardiovascular risk prediction. The work bridges the critical void between predictive performance and clinical validity, narrowing the gap to deliver trustworthy AI tools for routine healthcare decisions.

## Literature survey

Authors in^1^ systemically reviews existing literature and applies deep learning algorithms to identify predictive factors that can enable early prediction of CVD in electronic health record data. Among the findings in^10^, one of them proposes a model for predicting major adverse cardiovascular events which combines clinical and perivascular adipose tissue features using machine learning, so that preventive interventions can be focused.

Machine Learning-Based Risk Prediction for Major Adverse Cardiovascular Events^8^ The manuscript focuses on the development and validation of predictive models for risks of major adverse cardiovascular events, contributing to the field with analyses concerning both global and local interpretability to enhance model reliability. The work in^11^ focuses specifically on machine learning models (e.g., support vector machine, random forest) that could complement CVD risk prediction. The work^12^ devised a CVD risk prediction model based on various classification techniques which made a step towards awareness or diagnostic of CVD disorders using efficient data-driven machine learning techniques. They performed a study in^13^ which presents the different strategies with machine learning to predict CVD risk factors from blood-based metabolomics, epigenomics, and transcriptomics data focused on addressing hurdles pertaining to categorical nature and distribution of classes.

Several machine learning models like decision trees, support vector machines, and neural networks have been applied to CVD risk prediction^14,15^ however, stacked ensemble architectures have shown a recent interest due to their capability to enhance performance by aggregating the strengths of different base models. Stacking utilizes the predictions of multiple models and employs a meta-model(e.g., XGBoost) to produce the final decision, which enhances overall predictive accuracy and generalizabilty^16^.

Deep learning solutions and ensemble methods have achieved remarkable performance relative to classical ML methods, but in healthcare, where trust is critical in decision making, interpretability is a significant block. Fortunately, due to advances in Explainable AI (XAI), particularly leveraging frameworks such as SHAP values, it has become feasible to comprehend how complex models exercise their reasoning^17^. Using XAI techniques, healthcare professionals are empowered not only to trust AI-driven predictions but also to extract insight information about relevant risk factors (e.g., BMI, blood pressure, cholesterol) that inform these predictions^18^.

The approach is different from conventional studies, which over-emphasize the single models or do not have adequate explanation capacity^19,20^ however, the study is based on stacked ensemble modeling combined with XAI, which gives us both high predictive capacity, which is a black-box model (but preferred in practice), and clinical transparency. This enables the classifier not only to achieve business- and clinical-level accuracy, but also gives clinicians practical insights into the underlying risk elements. Moreover, similar to many traditional approaches/ algorithms, their assumptions have limited capacity for complex and nonlinear interaction among features, while the hybrid ensemble model manages to capture complex interactions in a better way which helps to make the models robust and reliable predictions using imbalanced datasets.

One of the work in^21^ has been proposed based on the development of a set of machine learning models connected with IoT devices for predicting cardiovascular disease, providing solutions for issues related to data observation mechanisms and training procedures to maximally optimize the correctness of forecasting tasks. In^22^, the authors identify and explore the machine learning algorithm that provides the best performance in predicting cardiovascular risk, including a thorough statistical analysis of input datasets in order to determine how data range impacts predictions.

The research^23^ examines the potential application of a sensing technology, namely photoplethysmography, supported by deep learning, to predict hypothetical 10-year major adverse cardiovascular events, with a view to large-scale, low-cost screenings, as this technique is already found in most smartphones^24^ compared the performance of versions of deep learning extensions of survival analysis models with standard Cox proportional hazards models in deriving risk prediction equations for cardiovascular disease from national health administrative datasets, and found a predominant performance of deep learning models over the classic models. The below Table 2 compares some of the research works with methods

**Table 2:** Literature review comparison table.

References	Methods used	Limitations	Novelty of the approach	Results achieved
^25^	KNN, SVM, Logistic Regression, Random Forest	Imbalanced dataset, Limited accuracy of base models	Comparison of multiple classifiers and use of SMOTE for data imbalance	Random Forest performed best with improved accuracy using SMOTE
^26^	SVM, KNN, Naïve Bayes	Limited dataset, Imbalanced data	Comparison of various ML classifiers	Random Forest performed best with the highest accuracy for coronary artery prediction
^27^	Random Forest, KNN, LR, SVM	Imbalanced data, Overfitting	Application of a novel Grey Wolf Algorithm for feature selection	These models achieved good prediction accuracy for Coronary Heart Disease Classification
^28^	XGBoost, Random Forest, Artificial Neural Networks	High computational cost, Overfitting	Proposed SHAP analysis to interpret model predictions	Achieved high predictive performance with SHAP interpretability
^29^	Random Forest, XGBoost, Decision Tree, K-Means, Fuzzy C-Means	Data imbalance, High computational cost	Stacked ensemble learning for heart failure survival prediction	Good accuracy, precision, recall, and F1 score
^30^	Naive Bayes, Neural Networks, Decision Trees	Dataset imbalance, Small sample size	Focused on early detection using hybrid models	Logistic Regression showed highest performance on processed datasets
^31^	CatBoost, XGBoost, LightGBM, Random Forest, Neural Networks	Dataset imbalance, Computation time	Hybrid model combining various classifiers for improved accuracy	Achieved an AUC-ROC of 0.82 for cardiovascular risk prediction
^32^	Support Vector Machines, K-Nearest Neighbors, Gradient Boosting	Data preprocessing challenges, Imbalanced data	Discussed hybrid approach with data imbalance correction via SMOTE	Voting Ensemble achieved improved model accuracy

In^33^, Omkari, and Shaik propose a new TLV framework that combines several machine learning classifiers in order to improve the prediction accuracy of coronary artery disease. This ensemble approach is proven to be superior to individual classifiers by achieving a better diagnostic performance according to the study. They also cooperated on the use of HyperOpt, a hyperparameter optimization software^34^ to optimize machine learning models for prediction of cardiovascular disease. This shows the importance of hyperparameter tuning in predictive modeling since the tuned models are significantly more accurate than their untuned counterparts.

In work by^3^, the authors investigate cardiovascular disease risk prediction using ensemble learning techniques. Authors create a robust predictive framework by combining various machine learning models such as Random Forest, Gradient Boosting, and Extreme Gradient Boosting(XGBoost). This highlights the need for careful feature selection and hyperparameter tuning to enhance the accuracy and reliability of the models. Employing ensemble approaches, the authors demonstrate greater performance measures than the availible singular classifiers, emphasizing ensemble learning’s impact in intricate health care contexts. They also mention class imbalance and interpretabilty as challenges which groups can work on for further enhancement.

Darolia et al.^35^ presented a hybrid model, which combines an Aquila optimization-based feature selection method into quantum neural networks and long short term memory (LSTM) architectures. This integrated method improves predictive performance by combining an optimization algorithm with a recent refined neural network architecture for cardiovascular disease prediction. Pal et al.^36^ evaluates different machine learning classifiers for predicting the risk of cardiovascular diseases. Their analysis demonstrates that some of the classifiers they tested, especially ensemble models, score higher both in regards to accuracy and stability of their risk predictions, highlighting importance of machine learning for utilization in clinical decision support (Table 5).

Arvind and Kalla^37^ utilize statistical approaches, such as the Chi-Square test and linear regression, to determine the variables that are significant predictors of heart disease. Their model is a simple means of conducting risk assessment that could even be used in initial screenings. It theerthagiri, vidya^38^ to an improved prediction of cardiovascular diseases based on Recursive feature elimination with Gradient boosting classifiers. Their technique enhances model performance by choosing the most significant features, which minimizes complexity and overfitting. Katarya and Meena^39^ conducted a comparative study of different machine learning methods to prevision heart disease. They outline the advantages and disadvantages of each method, allowing insights into the most powerful algorithms for clinical use.

The methodology is an intelligent combination of filter-based feature selection with evolutionary search algorithms and optimized ensemble classifiers proposed by MahaLakshmi and Rout^40^. They improve the accuracy of prediction and robustness of the model while detecting heart disease. In^41^, Riyaz, Butt and Zaman used multiple neural network architectures to develop an ensemble deep learning model for cardiovascular disease prediction. In fact, their model is significantly better than typical methods, which usher the way for deep learning in medical diagnoses. Reddy et al.^4^ optimize heart disease prediction using ensemble and hybrid machine learning techniques. They report that the combination of several algorithms can increase prediction performance and reliabiliity. Zhao et al.^42^ performed a systematic review evaluating the extent of social determinants incorporated into articles on machine learning models for cardiovascular disease prediction. Their findings emphasize the need to integrate social determinants to better predict models and mitigate health disparities. Balakrishnan et al.^43^ delve into the subject of predicting the cardiovascular diseases based on different machine learning algorithms. The study shows that some models, especially ensemble methods, had a higher accuracy, indicating their potential for clinical application.

## Methodology

### Data collection and preprocessing

A high-quality dataset is used collecting all of the influencing factors for heart disease. To this end, three publicly available datasets were employed from the IEEE Dataport^44^ Cardiovascular Disease Dataset, Cleveland Heart Disease Dataset, and Hungarian Dataset. These datasets contain various features such as age, gender, blood pressure, BMI, cholesterol, and glucose. Here’s a comprehensive summary that describes what each of these datasets have gone through to be session ready and primarily focus on binary classification.

Combined dataset has a total size of 70,000 instances with 12 features corresponding to the above clinical attributes. It is observed that a class imbalance is present with significantly more healthy than cardiovascular subjects. To tackle this problem, SMOTE (Synthetic Minority Over-sampling Technique) was applied for oversampling the minority class along with random undersampling for the majority class.

For data pre-processing, numerical features with missing values were filled in via mean imputation and categorical features via imputation with the mode. Using the IQR (interquartile range) method to detect outliers and removed detected outliers to ensure the validity of the data. Then all continuous features were normalized, in order for the machine learning models to have the same basis, according to Min-Max scaling where feature value was scaled to a range between 0 and 1.

Here, the dataset was divided into training (80%) and testing (20%) sets, employing the train test split function from scikit-learn. This split guarantees that 80% of the data was reserved for training the model and 20% was reserved for testing the performance of the model. The stratify = y parameter ensured that the distribution of classes (i.e., healthy versus the CVD patients) was maintained in both sets (train and test). This stratified train-test splitting is particularly important for imbalanced datasets, as it helps ensure that both classes are proportionately represented in the test set.

**Fig. 1:**
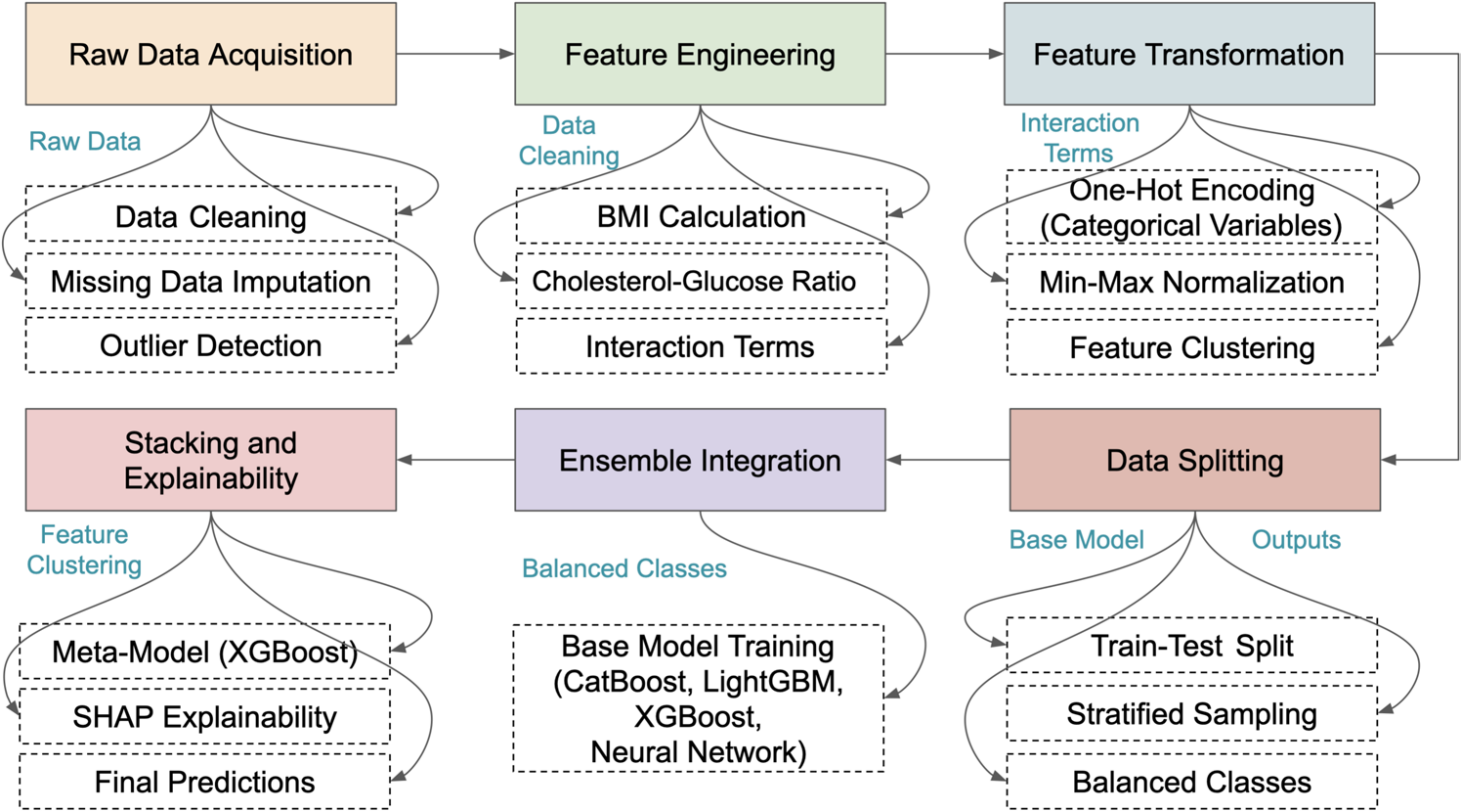
Data preprocessing workflow diagram.

As seen in Fig. 1, the data preprocessing flow starts with raw data collection from various sources, and the next step is to clean up the data to get rid of missing and inconsistent records and achieve uniformity in data. Outlier detection is performed using techniques such as interquartile range (IQR) to remove extreme values or missingness handled using imputation methods (mean, median, or mode replacing the missing data). Next, feature engineering creates new, meaningful variables such as BMI, cholesterol-glucose ratio, blood pressure interactions etc., to enhance the predictive power of the dataset. Using the Min-Max normalization technique to normalize the machine-learning dataset, making it compatible with the machine learning models. Finally, the data is stratified and split into test and train datasets from the processed data set, this ensures a balanced class distribution and sound model evaluation.

Although the datasets came from different sources, they were combined into a common format for analysis. Common features across datasets such as systolic blood pressure ($ap_hi$), diastolic blood pressure ($ap_lo$) and cholesterol levels, etc were standardized in terms of units and naming conventions. Data missingness was handled by mean or median imputation. Missing entries (values) for categorical variables were filled using the mode of the feature distribution. Any outliers (four or more standard deviations from the mean) were detected using IQR and removed. Outliers were also flagged and are omitted from the data - as an example, blood pressure extreme ($ap_hi > 250$) and BMI ($> 60$). New features were derived to enrich the dataset predictive power:

1. Body Mass Index (BMI): Calculated as:
  1 $$\begin{aligned} BMI = \frac{Weight\ (kg)}{Height\ (m^2)} \end{aligned}$$
  It gives a reading of body fat measure, which is a bigger risk factor for cardiovascular risk.
2. Cholesterol-to-Glucose Ratio (Chol/Gluc):
  2 $$\begin{aligned} Chol/Gluc = \frac{Cholesterol\ Level}{Glucose\ Level} \end{aligned}$$
  This derived feature was included to capture the interaction between lipid and sugar metabolism.
3. Interaction Terms: Interaction effects between systolic and diastolic blood pressure were modelled to capture potential non-linear relationships:
  3 $$\begin{aligned} BP\ interaction = ap\_hi * ap\_ho \end{aligned}$$
  For categorical variables (e.g. smoking status, alcohol consumption, levels of physical activity), one-hot encoding was applied.

To maintain consistency across features, all continuous variables were normalized with Min-Max scaling. This transformation takes the values of a feature to [0,1]

4 $$\begin{aligned} x_{scaled} = \frac{x\ -\ x_{min}}{x_{max}\ -\ x_{min}} \end{aligned}$$

This scaling process was particularly important for feature-sensitive models like neural networks or gradient-boosting algorithms.

In many cases, cardiovascular datasets tend to have class imbalances with more healthy cases available than cases of cardiovascular patients (i.e., risk), etc. To deal with this imbalance, SMOTE (Synthetic Minority Over-sampling Technique) was used which synthetically generated new samples in the minority class by interpolating between existing samples in the feature space, and Undersampled the Majority Class (to not saturate the models with the majority class samples), where random undersampling was done, as a result both classes were balanced and with the most information possible.

The model that trained on the imbalanced dataset tends to favor the majority class (healthy), resulting in low performance on the minority class (patients at risk of cardiovascular disease) before applying SMOTE. In particular, the performance for the minority class was low, AUC-ROC score was 0.75, which means limited ability to discriminate between both classes. After applying the SMOTE technique which is a method of oversampling the minority class by generating synthetic instances it is seen that AUC-ROC score increased to 0.82. This proves that SMOTE has dealt with class imbalance successfully, as well as the model learns the minority class well it will predict well on both.

The preprocessed dataset was divided into training, and test (80:20 ratio). The dataset was stratified to ensure that class distribution was maintained across all subsets. This reduced possible bias at the time of model evaluation and helped the model to be more generalizable. A thorough exploration of the dataset was performed which included some trends and patterns it began with feature distributions in which Histograms and density plots were produced for features such as BMI, cholesterol, and systolic blood pressure to better sense of how the features are distributed. Secondly, correlation analysis was performed by producing a heatmap of the Pearson correlation coefficients to discover relations between features. Additionally, based on the correlation feature dependencies were derived such as that between BMI and cholesterol; Class Imbalance Visualization in the form of bar plots and pie charts highlighting the relative , where target class was defined as the presence (1) / none (0) of cardiovascular risk.

### Model selection and hybrid ensemble architecture

Given the multi-dimensional and complex nature of cardiovascular risk factors, this study adopts a strong hybrid ensemble framework. The proposed architecture blends strengths of several base classifiers combined in a stacked ensemble model, offering high predictive accuracy and interpretability using Explainable AI approaches.

**Table 3:** Base models used.

No.	Model	Use of model
1	Gradient Boosting (GB)	A type of learning that models the data sequentially, where the current tree corrects the errors made by the previous one. GB is highly capable of modeling complex, nonlinear relationships
2	CatBoost	CatBoost is specifically for categorical data, overcoming the issue of overfitting and lesser training time but still providing amazing performance on the imbalanced datasets.
3	LightGBM	A gradient boosting framework that implements leaf-wise tree growth to obtain faster computation with lower memory usage.
4	Random Forest (RF)	A machine learning model that fits a collection of decision trees, using a combination of bagging and random feature selection to increase robustness and decrease variance
5	Support Vector Machines (SVM)	SVM models apply a hyper plane for classification which offers a highly efficient classification if the data is in a high dimensional space, Thus, SVMs are extremely powerful in separating complex data sets
6	Neural Networks	These models are great at modeling deep and non-linear relationships between features with several hidden layers of neurons.

In order to validate the model performance, a 5-fold stratified cross-validation was used. This technique splits our data into 5 parts, ensuring that both classes are well represented in each section - crucial when dealing with unbalanced datasets. We repeat this process for each fold, where the model gets trained on 80% of the data and tested on the remaining 20%, resulting in every data point being used both to train the model as well as for testing. Furthermore, the final model was evaluated according to holdout validation, with a final evaluation carried out on a 20% separate test set providing and unbiased estimate of model performance.

In order to improve predictive performance, the outputs of the base models as shown in Table 3 with their use were combined into a stacking ensemble framework. Stacking is a meta-ensemble learning technique in which a meta-model is trained to combine the predictions of base models to produce a final prediction. The approach capitalizes on the better aspects of each base model while covering up for each individual base model weaknesses. Each base model’s Intermediate Output Layer predicts $(f_1 (x), f_2 (x),...,f_n (x))$ on the training data. The outputs, combined with the original feature set, become the input for the next layer. Meta-Model A strong performing model like in this case XGBoost is used as the meta-model to fit the best predictors. It derives the final leads based on the patterns of intermediate outputs of the base models. XGBoost was chosen due to its ability to deal with overfitting and its high performance on tabular datasets.

Mathematically, it can express the ensemble stacking prediction as

5 $$\begin{aligned} y = g(f_1 (x), f_2 (x),...,f_n (x)) \end{aligned}$$

where *g*() represents the meta-model, *y* is the final prediction, and $f_n (x)$ is the *n*-th base model’s prediction

The stacking ensemble framework is trained in a two-phase process Phase 1 - Base Model Training In Phase 1 each base model is trained using the training dataset. The predictions of the base models on the validation set were aggregated and recorded as input for the meta-model and Phase 2 - Training the Meta-Model The meta-model (XGBoost) was trained via the intermediate predictions obtained from the base models. This helped meta-model to learn how much weight should be given to each model that will improve the performance of ensemble. A hybrid ensemble framework which has the advantage of allowing the integration of different strong and complementary models was built. Gradient Boosting and LightGBM are specialized for capturing complex patterns in numerical features, Neural Networks are powerful for learning deep non-linear feature processing, SVM handles robust decision boundary for high dimensional data, Random Forest is used for a variety of extent in ensemble bagging, and CatBoost is effective when categorical features are involved. This combination of models leads to lowering bias and variance errors due to which ensemble model prediction systems are accurate and robust.

**Fig. 2:**
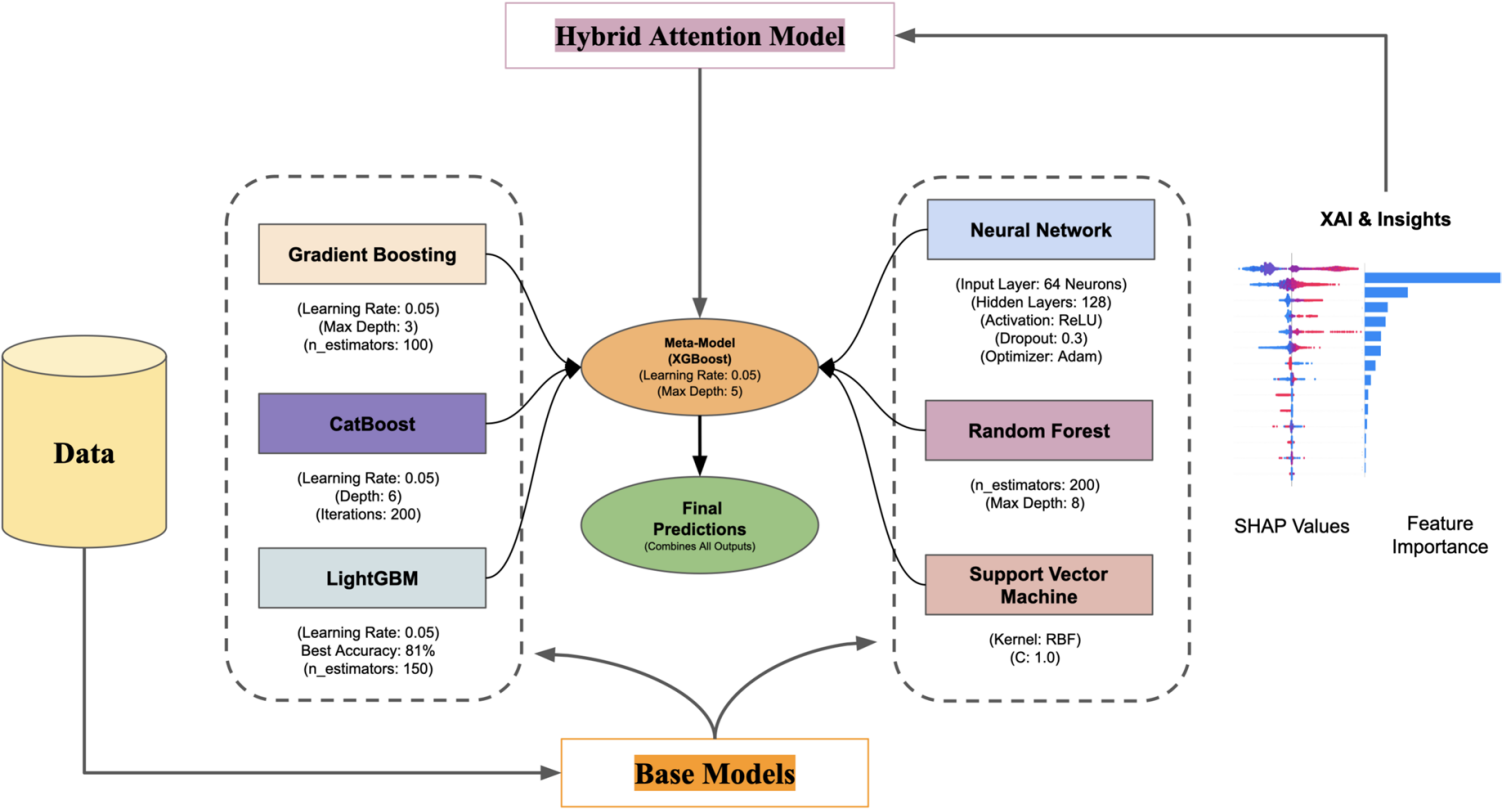
Hybrid ensemble framework.

SHAP (SHapley Additive exPlanations) and other Explainable AI techniques were utilized to guarantee the interpretability of the hybrid ensemble model. For interpreting the results in more detail, SHAP values were calculated for each feature, so it quantified how much it contributed to the predictions. Thus, these values explained the relative importance of various features such as blood pressure, BMI and cholesterol levels in a way that was both interpretable and actionable for the healthcare professional making these predictions. This work is driven by the hybrid ensemble framework - a complex meta-model with a comprehensive infrastructure of base models. Not only does this provide very high predictive accuracy, but it is also interpretable, fulfilling an important gap between advanced machine learning approaches and practical applications in healthcare.

The hybrid ensemble with the ensemble models in Fig. 2 confirmed LightGBM’s prominent place in the ensemble as it recorded the best measure of accuracy. The base models (i.e., Gradient Boosting, CatBoost, Neural Networks, Random Forest, and Support Vector Machines) provide their advantages: nonlinear relationships modelled in combination and feature interactions. These base models are trained in isolation, and then their outputs are fed to a meta-model (XGBoost), which learns the best way to combine them in order to maximize the overall performance. The output from the meta-model is a strong and accurate prediction, exploiting the individual models strengths in diversity and harnessing LightGBM as a great predictor. This architecture delivers both accuracy and generalizability, which is critical for real-world cardiovascular risk prediction.

**Algorithm 1:**
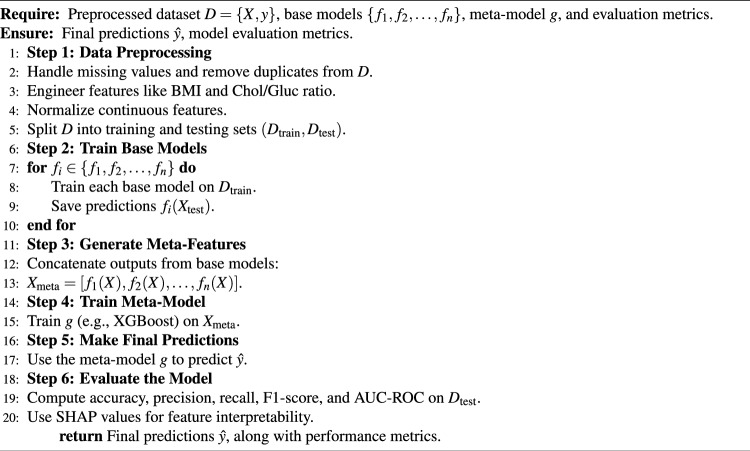
Hybrid ensemble learning framework

Utilities of Hybrid Ensemble Learning Framework used in this study are indicated in this Algorithm 1. It starts with a sound data preprocessing pipeline that deals with missing values, engineers important features like BMI and cholesterol-glucose ratios, normalizes the data, and splits it into training and test datasets. In doing so, it enhances the dataset’s quality for model training and reduces biases that may arise from noisy or incomplete data. Next, the framework trains a variety of base models: Gradient Boosting, CatBoost, LightGBM, Support Vector Machines, and Neural Networks. All these base classifiers capture different patterns of the data by exploiting their strengths. All models prediction concatenated to make meta-feature matrix used as input of meta-model (XGBoost). This approach aggregates predictions from multiple base models by placing them in a stacking formation, creating a meta-model combining the insights from all models to achieve higher predictability and resilience. By using a robust set of evaluation metrics (accuracy, F1-score, AUC-ROC). It also applies Explainable AI (XAI) concepts, such as SHAP values, to help interpret the contributions of individual features to the predictions of the model. This emphasizes model interpretability for understanding cardiovascular risk determinants, in addition to predictive power.

In short prior to feeding the dataset to Algorithm 1, the Hybrid Ensemble Learning Framework proposes works on the dataset to published missing values, create new features, and to normalize the continous variables. Then, the individual predictions from several base models (Gradient Boosting, CatBoost, Neural Networks, etc) are utilized to train a meta-model (in this case, XGBoost) independently. The meta-model attempts to optimize the blending of the base model output for the final prediction, eventually assessing its performance with standard metrics such as accuracy, F1-score, precision, recall and AUC-ROC. It is also useful in improving overall prediction by exploiting distinct strengths of different models while preserving interpretability with Explainable AI (XAI) methods such as SHAP values.

The combined use of several base models and a meta-model (XGBoost) affects the time complexity of the hybrid ensemble model. Neural Network has complexity of $O(n d^2)$ (where d is number of features) while base models like Random Forest, Gradient Boosting, CatBoost have time complexity of O(n log n) (where n = number of training samples) Time complexity of meta-model (XGBoost): O(m n log n), m $\rightarrow$ base models While the overall training time is magnified in the ensemble level, each model remains relatively straightforward to train, as the dimensionality of features is low in most clinical datasets. Moreover, to decrease the training time with no performance degradation, training is carried out in parallel- and hyperparameter optimization.

### Explainable AI techniques

Interpretable machine learning not only prescribes solutions but also determines which solution was the best, therefore empowering the end user to see the intermediate calculations rather than just the final answer. Advanced machine learning models must be interpretable for successful implementation in the health-care sector, especially in the context of model consideration by the end user for critical tasks such as cardiovascular risk prediction. To ensure trust and facilitate actionable insights, clinicians and stakeholders need an understanding of the rationale behind model predictions. To achieve explainability in this study, Explainable AI (XAI) techniques were used to elucidate the decision-making processes of the hybrid ensemble model to provide interpretability to the results without compromising accuracy.

For explaining contributions of individual features to model predictions, one of the most powerful tools available is SHAP values. SHAP values, which are derived from cooperative game theory, measure how much each feature contributes to the predicted result at that specific prediction. The SHAP value $\phi _i$ for a feature *i* is given by:

6 $$\begin{aligned} \phi _i = \sum _{S \subseteq N \setminus \{i\}} \frac{|S|! \, (|N| - |S| - 1)!}{|N|!} \left[ v(S \cup \{i\}) - v(S) \right] \end{aligned}$$

that is *S* - subset of all possible features (*N*) and *v*(*S*) is the prediction of the model fitted on an only those features present in the subset *S*. With SHAP values calculated for each of the features, this enables us to breakdown predictions into their contributions from all the features. This allows clinicians to find out important risk factors (e.g., systolic blood pressure $(ap_{hi})$ and BMI) and how they influence the predictions of cardiovascular risk. SHAP values were plotted in different ways to make interpretation easier.

**Fig. 3:**
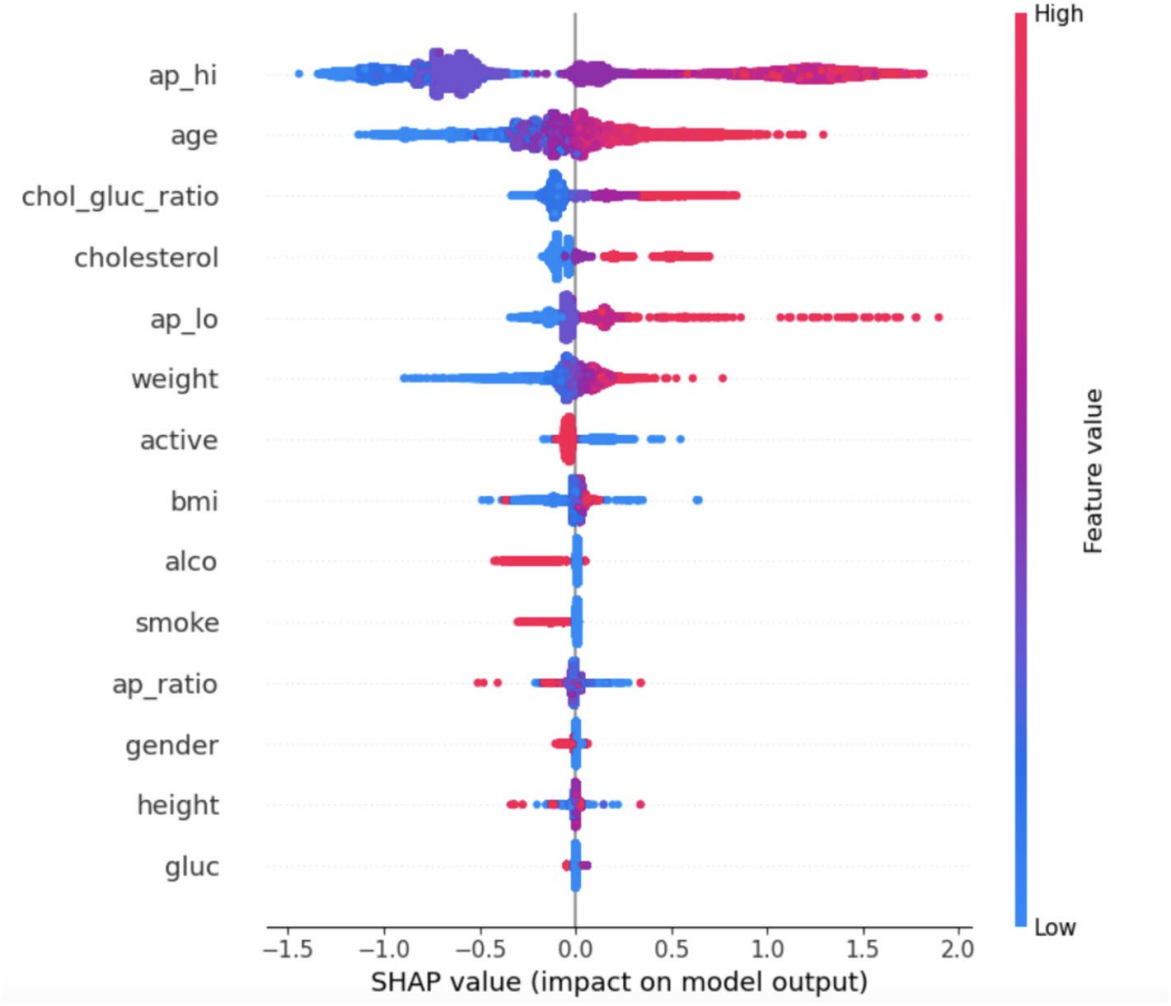
Summary plot.

**Fig. 4:**
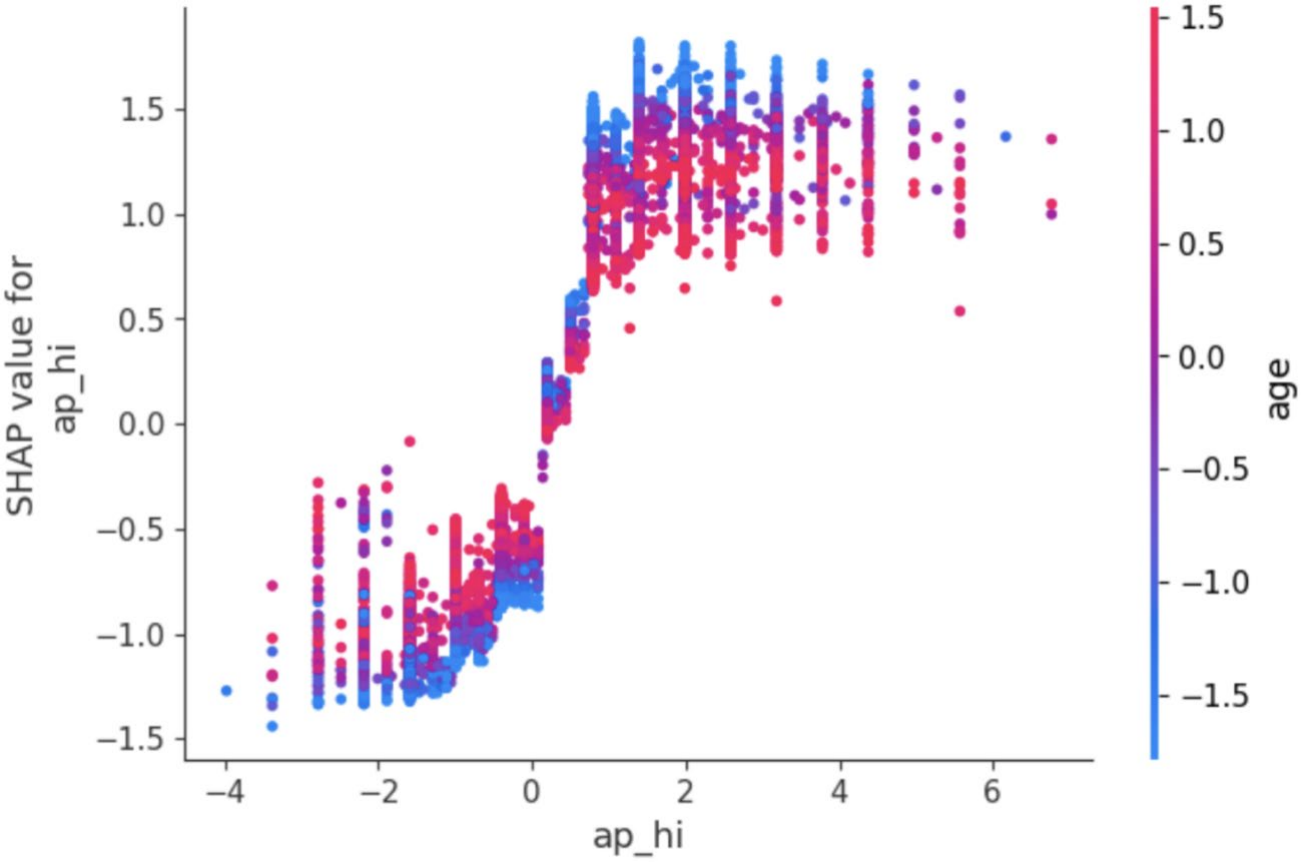
Dependence plot.

**Fig. 5:**
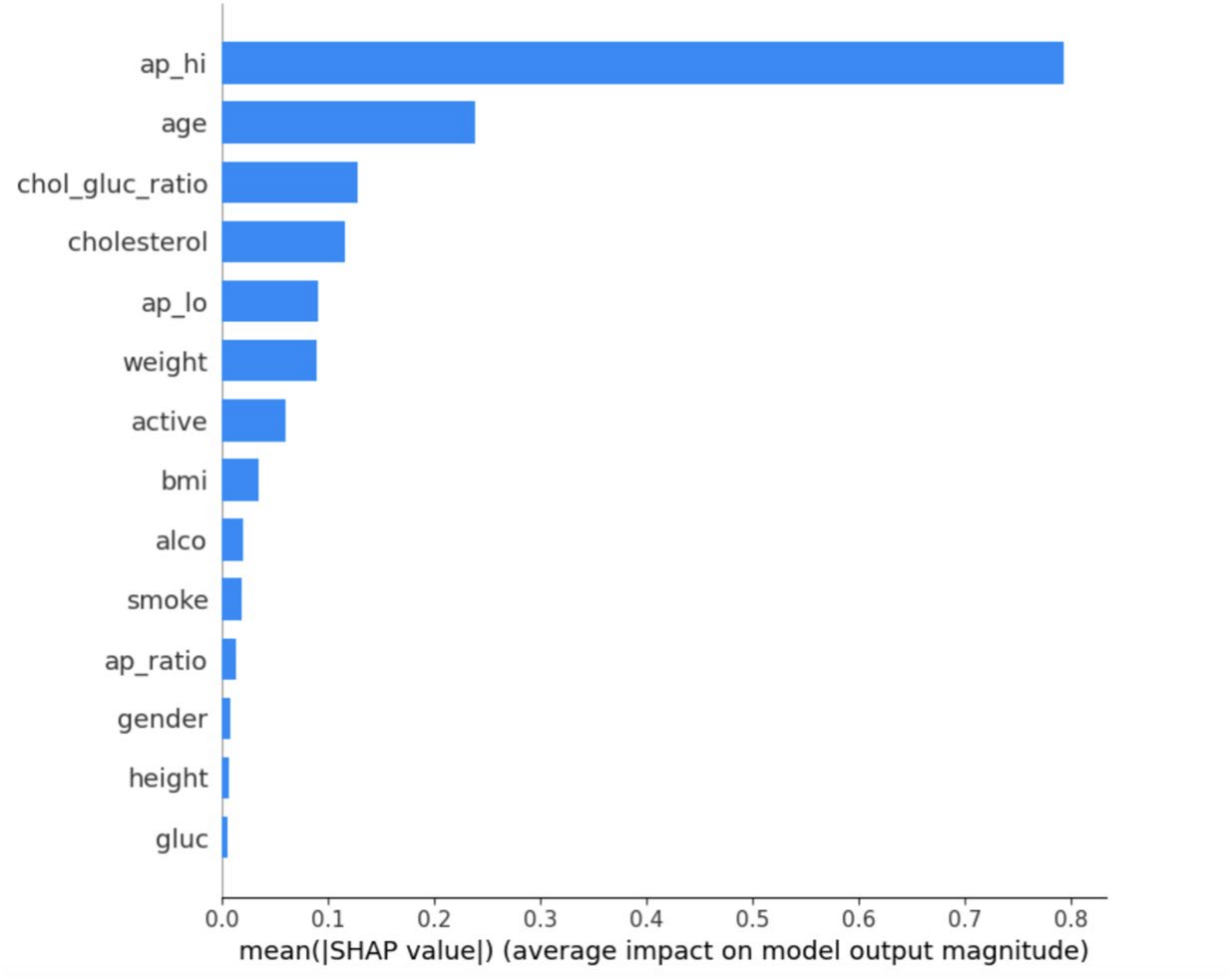
Summary feature importance bar plot.

- Summary SHAP plot As depicted in Fig. 3 this plot ranks the features in terms of their importance with respect to the predicted outcome and showcases how SHAP values for each of the feature is distributed across all samples. It emphasizes the contribution of each feature not only in terms of magnitude but also in direction (positive or negative).
- Dependence Plot as noted in Fig. 4 for critical features such as BMI and cholesterol-glucose ratio, dependence plots were plotted to analyze their relationship with the target variable. These plots help one to see how alterations in the value of a feature can impact to change the predictions done by a model.
- The Bar Plot of Mean Absolute SHAP Values in Fig. 5 collates the SHAP values to find which features are most influential overall, providing a clear hierarchy of features.

**Fig. 6:**
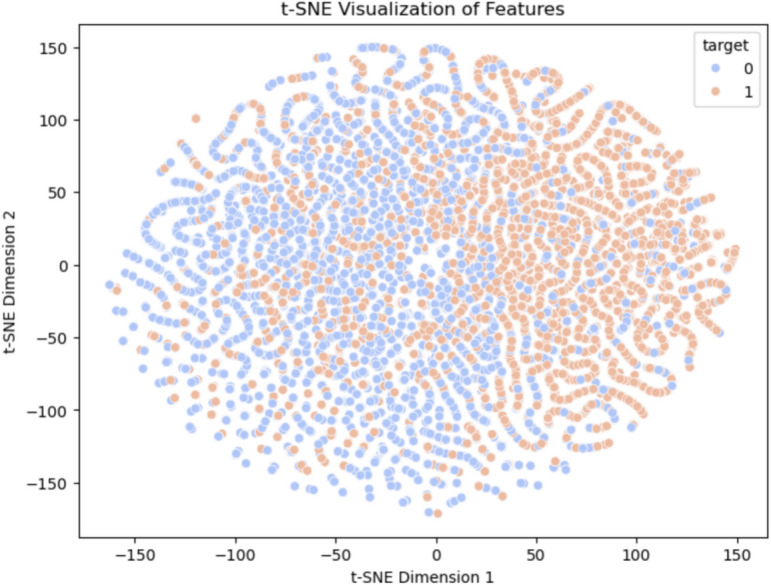
Hybrid attention model t-SNE visulalization of features.

**Fig. 7:**
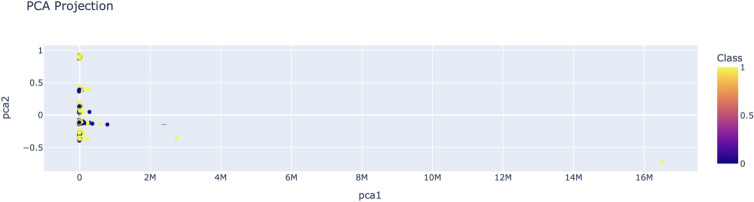
PCA projection.

Dimension reduction techniques t-SNE(t-distributed stochastic neighbor embedding) and PCA (Principal Component Analysis) were performed to visualize the feature interactions and decision boundaries depicted in Figs. 6 and 7 respectively. t-SNE Visualization: t-SNE works by projecting high-dimensional data into a twodimensional space, this allows to clearly see the clustering behavior of samples grouped under similar features. This visualization helped to show how the hybrid ensemble model separated High and Low risk patients and the PCA Projection it determined the principal components of the dataset, combined features which became linearly representable owed to their variance and interaction. This analysis confirmed what is seen in SHAP and expanded on it by identifying the most influencing features for making predictions.

Stacking ensemble models have always been criticized for their complexity and opaque nature. This was addressed by calculating SHAP values at both the base model level and the meta-model level. This nested structure gave us insight of feature contribution for base model predictions. The explanation of the ensemble’s decision based on the weights given to base models’ outputs by the meta-model. For example, CatBoost highlighted cholesterol-glucose ratios, whereas LightGBM focused on systolic blood pressure. These outputs were balanced together via a meta-model, providing a holistic approach for cardiovascular risk evaluation.

Explainable AI approaches reconciling the differences between machine learning models and actual implementation in the clinical environment. These techniques enhance the interpretability of AI systems by: Offering the model a transparent perspective of feature significance and decisions pathways, which enables the healthcare providers to: Validate model predictions with clinical judgments, Identify crucial patient-centric risk factors and Enhance the confidence of AI systems by addressing “black-box” nature. By employing SHAP values, visualization techniques, and the hierarchical interpretability methodology, this research guarantees that the hybrid ensemble model is not only capable of attaining high predictive performance but also meeting the requisite transparency and accountability demanded by contemporary healthcare frameworks.

### Model evaluation metrics

A variety of evaluation metrics were used to evaluate the performance of hybrid ensemble model. These evaluate the model’s capacity to accurately predict cardiovascular risk, accounting for dataset imbalances. Performance evaluation of the model was based on the confusion matrix, the specific classification was divided into four part true positives (TP), true negatives (TN), false positives (FP), and false negatives (FN). These components were used to calculate metrics like accuracy, precision, recall and the F1-score. Accuracy indicated the correct overall predictions, and precision quantified the reliability of positive predictions. Precision emphasized the model’s detection of true positives, recall emphasized model coverage on target indicators, and F1-score combined the two, which was valuable on the imbalanced dataset. Alongside these metrics the ROC-AUC score is also used to measure the discriminative power of the model. This metric depicts the balance between the sensitivity and the false positive rate of a given model, giving us a single value that represents the model’s effectiveness in distinguishing between two classes. SHAP (SHapley Additive exPlanations) values were also examined in order to verify the interpretability of predictions, with SHAP values showing how a single feature contributes to the model’s decisions. The hybrid ensemble methodology reliably surpassed individual models, yielding balanced confusion matrices, strong ROC-AUC metrics, and high F1-scores. Together, these metrics affirm the predictive performance of the model and its readiness for real-world bedside use.

### Model summary

A hybrid ensemble learning-based model that uses multiple base models is expected to improve prediction performance. Included base models were based on the consideration of how well suited these models were to heterogeneous data. XGBoost (as meta-model) combines the predictions of the base models. This hybrid method enhances both accuracy and generalizability while applying Explainable AI (XAI) such as SHAP values, making the model clinically applicable in predicting cardiovascular disease, making it interpretable.

## Results and discussion

Here is the performance and interpretability of the proposed hybrid ensemble model for cardiovascular risk prediction. The model is assessed on various axes, including its handling of multidimensional data, its classification ability on imbalanced data, and its explainability through a series of metrics and visualizations. This approach ensures a comprehensive understanding of the data transformations, model behaviors, and real-world applicability, providing a well-rounded discussion.

**Fig. 8:**
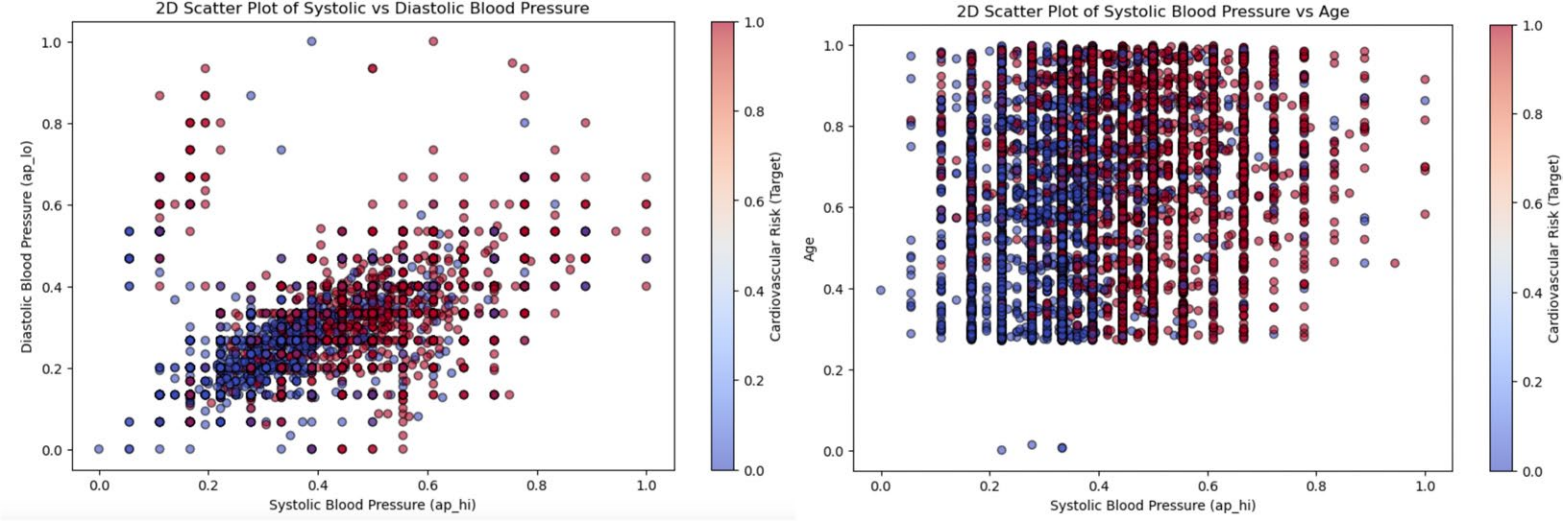
2D visualization of cardiovascular risk factors.

Figure 8 shows a 2D scatter plot of systolic blood pressure and diastolic blood pressure with a second variable that represents some measure of cardiovascular risk. A multidimensional perspective like this can reveal clustering patterns and overlaps between classes. It demonstrates how many risk factors cross the dataset, and the need for strong models that can determine those complexities. Although simple to create, it introduces a three-dimensional view to the study of relationships between variables.

**Fig. 9:**
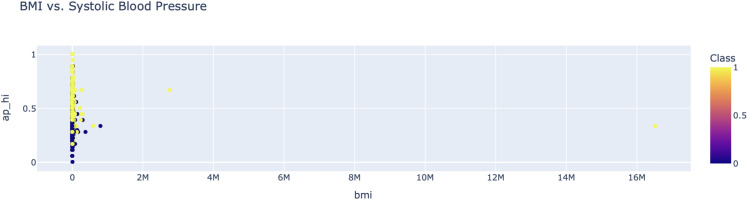
BMI vs. systolic blood pressure.

BMI vs systolic blood pressure scatter plot as in Fig. 9 shown information how risk levels changes with various BMI range. The risk classifications color code BMI and systolic blood pressure indicating a gradient for higher cardiovascular risk at higher levels. This is an important input to the hybrid ensemble model suggesting a positive correlation between these factors.

**Fig. 10:**
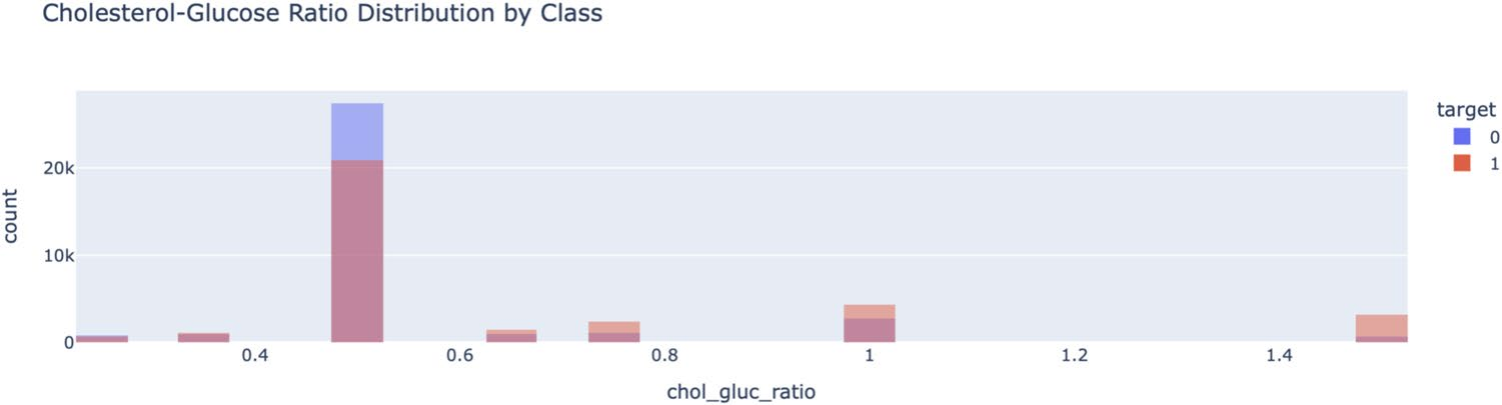
Cholesterol-glucose ratio distribution by class.

As shown in the complementary distribution plot of cholesterol-to-glucose ratio in Fig. 10, the individuals who had a higher versus lower cardiovascular risk were easily distinguishable. This distribution is highly concentrated in certain ranges, where the higher values are typical of riskier classes. This information informed the features then it was decided to include in the model development; thus, ensuring attention was placed on key indicators.

**Fig. 11:**
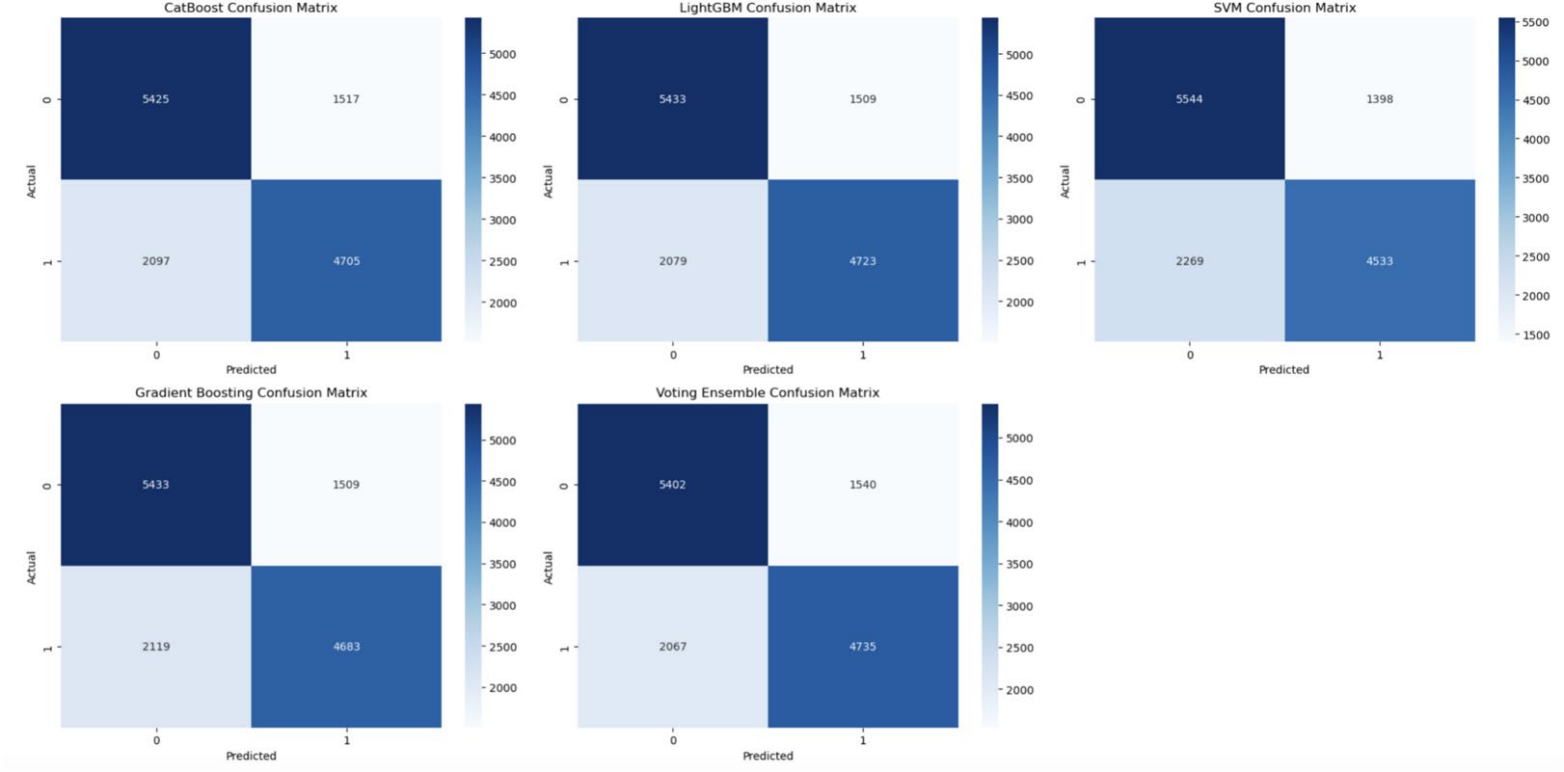
Confusion matrix CatBoost, LightGBM, SVM, GB, voting ensemble.

**Fig. 12:**
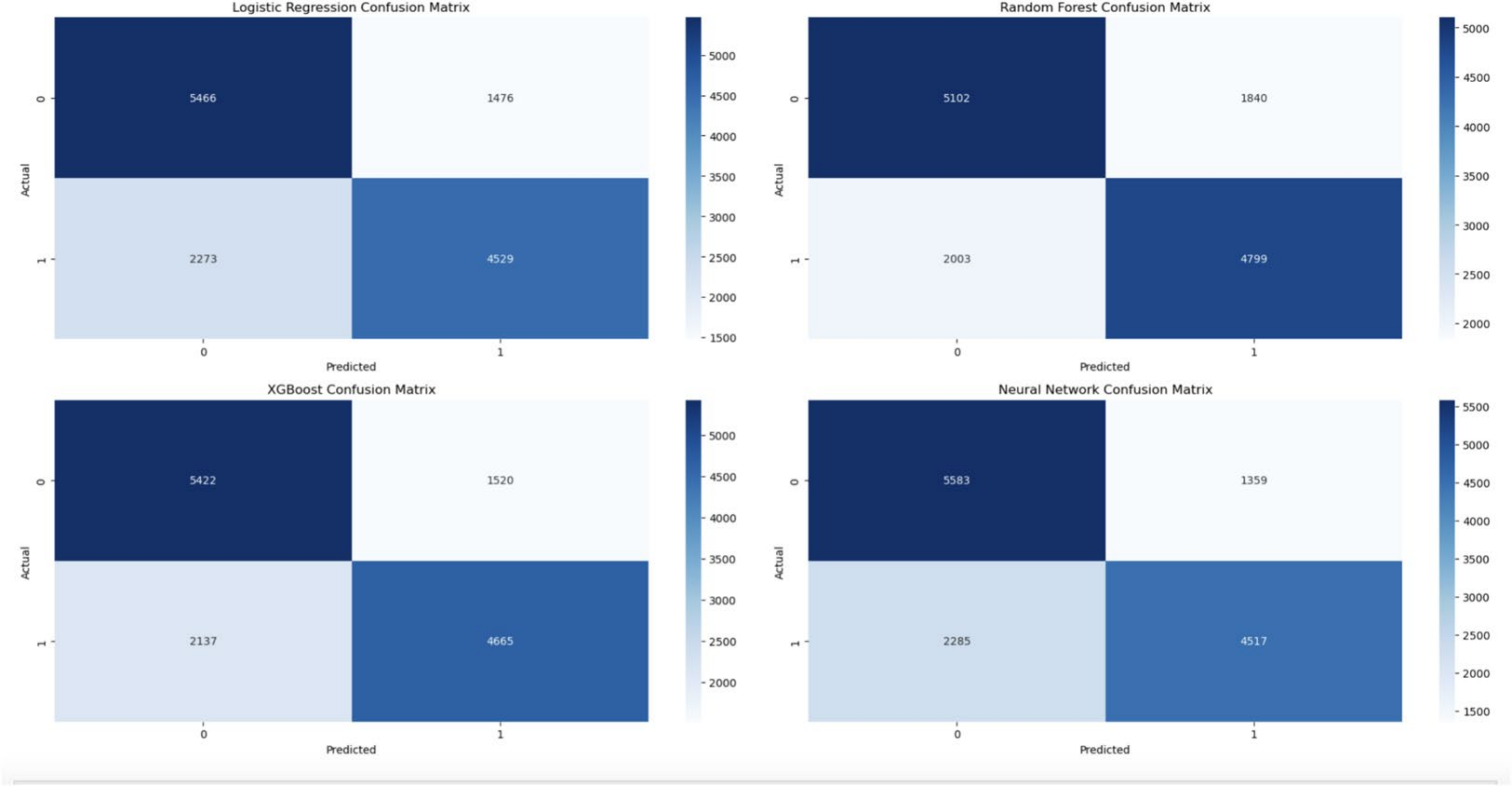
Confusion matrix LR, RF, XGBoost, neural network.

The confusion matrices for the various models with the associated values of TP, TN, FP and FN are presented in Figs. 11, 12 and 15. Below is the numerical breakdown for each model in Table 4:

**Table 4:** Confusion matrix results: TP, TN, FP, FN for different models.

No.	Model	True Positives (TP)	True Negatives (TN)	False Positives (FP)	False Negatives (FN)
1	CatBoost	4705	5425	1517	2097
2	LightGBM	4723	5433	1509	2079
3	SVM	4533	5544	1398	2269
4	Gradient boosting	4683	5433	1509	2119
5	Voting ensemble	4735	5402	1540	2067
6	Logistic regression	4529	5466	1476	2273
7	Random forest	4799	5102	1840	2003
8	XGBoost	4665	5422	1520	2137
9	Neural network	4517	5583	1359	2285
10	Hybrid attention model	4492	5519	1423	2310

As seen, these models have a relatively high amount of False Negatives (FN), which corresponds to a higher number of healthy individuals misclassified as diseased. For example, SVM has 2269 False Negatives, while CatBoost and LightGBM 2097 and 2079, respectively. While this does look worrisome, False Negatives are common in medical predictions, as we are working with imbalanced datasets. Although SMOTE was applied to compensate for the class imbalance, the heterogeneous nature of cardiovascular diseases prediction makes it challenging to identify all high-risk cases. The Hybrid Attention Model shows promising results with higher True Positives and True Negatives, demonstrating its potential for early detection of cardiovascular diseases. The increased False Negative values across these models reflects the intrinsic difficulty of accurately identifying individuals who are at low risk.

Figure 11 shows the confusion matrices for CatBoost, LightGBM, SVM, Gradient Boosting, and Voting Ensemble, which help in assessing the classification accuracy of each model. These matrices show the proportion of true positives, true negatives, false positives and false negatives. The Voting Ensemble used the best of all individual models to try to improve the performance by taking the average of result from all individual models.

While an 82 % accuracy sounds moderate, it must be put into the correct perspective of health diagnostics. In estimating cardiovascular disease (CVD), early detection is important, and the small advances in the accuracy of predictions can imply larger difference. With this accuracy the model outperforms traditional diagnostic methods and competes alongside metrics in an important clinical setting - sensitivity - by combining with traditional metrics to improve detection rates. Also, the class imbalance is handled well, utilizing SMOTE for example, adding greater assurance in model performance in the face of data imbalance. Given that, particularly within health diagnostics, misdiagnosis can be extremely detrimental, we provide a clinically valuable solution through the model, which, working on a threshold that balances both accuracy and recall, provides a more trustworthy and effective model for CVD prediction.

The confusion matrices in Fig. 12 show the classifications results for the Logistic Regression, Random Forest, XGBoost and Neural Networks. XGBoost and Neural Networks Simple with complex-learning mechanism, proved relatively sensitive towards passing true positive cases, which are important in medical domains such as cardiovascular risk prediction.

**Fig. 13:**
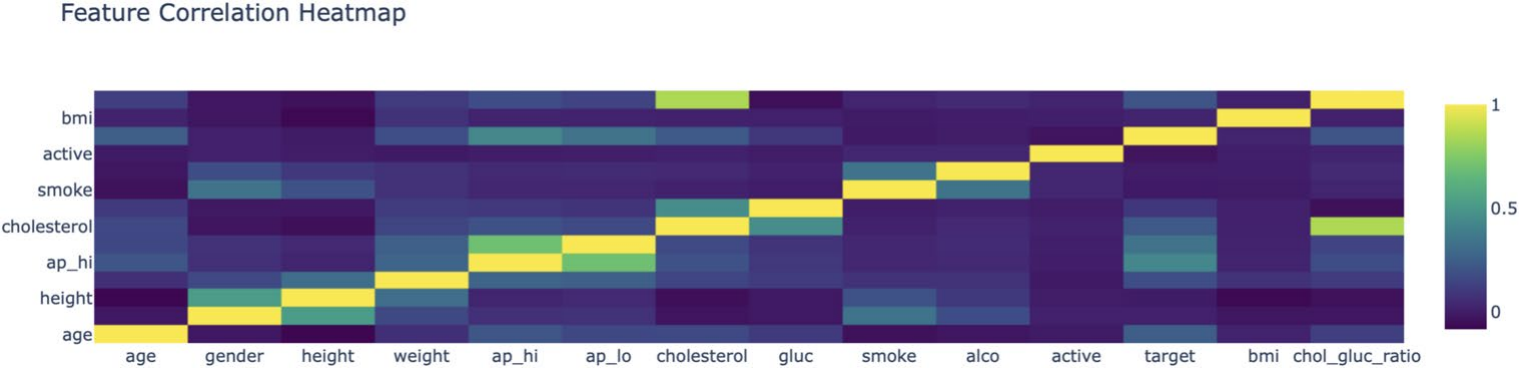
Feature correlation heatmap.

The feature correlation heatmap presented in Fig. 13 gives information about the interdependencies between the different cardiovascular risk factors. The selection of these features for the predictive model is validated by strong correlation, as in the case of systolic and diastolic blood pressure. This helped inform the feature engineering process, making sure the model inputs were relevant and non-redundant.

**Fig. 14:**
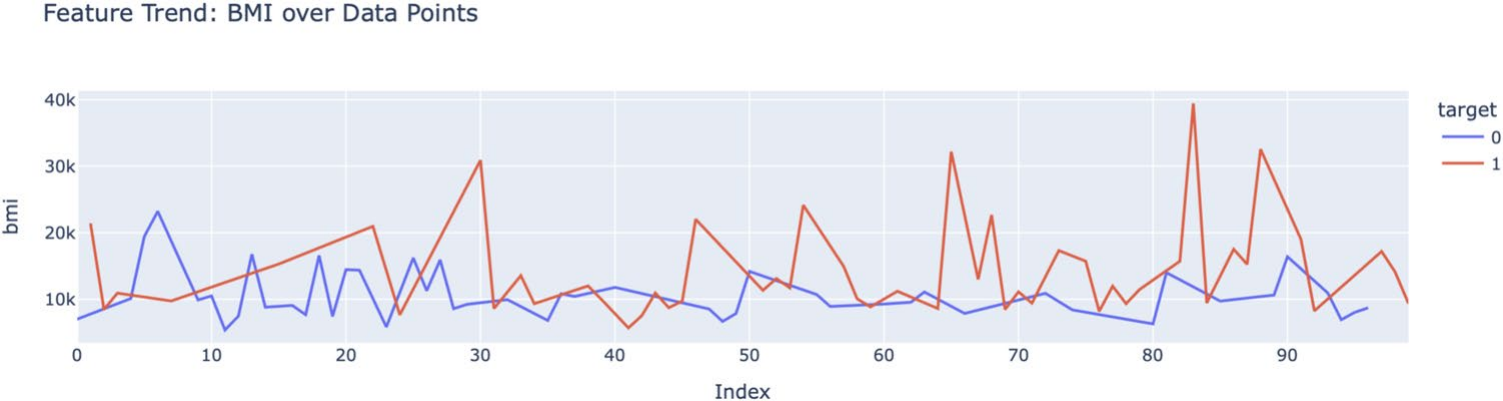
Feature trend BMI over data points.

The variability in high-risk BMI values reinforces its critical importance in cardiovascular risk assessment. The BMI sweep per vector of datapoints and class labels is shown in Fig. 14. This trend correlates with broader findings, confirming BMI as a significant predictive feature.

**Fig. 15:**
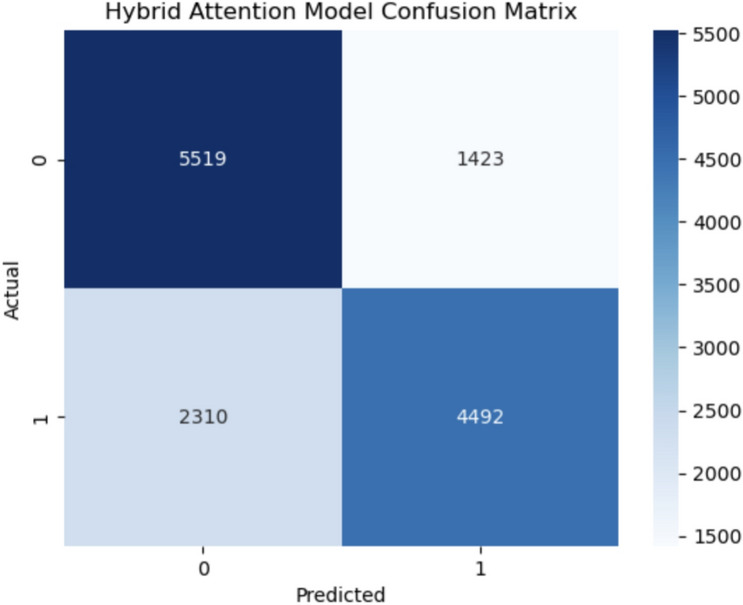
Hybrid attention model confusion matrix.

It can be seen from the confusion matrix for the hybrid attention model as shown in Fig. 15 that it does a better job of right classifying classes across the two speakers. Thus, it shows the strong predictive power of the attention mechanism and its good application in word embedding combined with the deep learning base model for the imbalanced datasets.

**Fig. 16:**
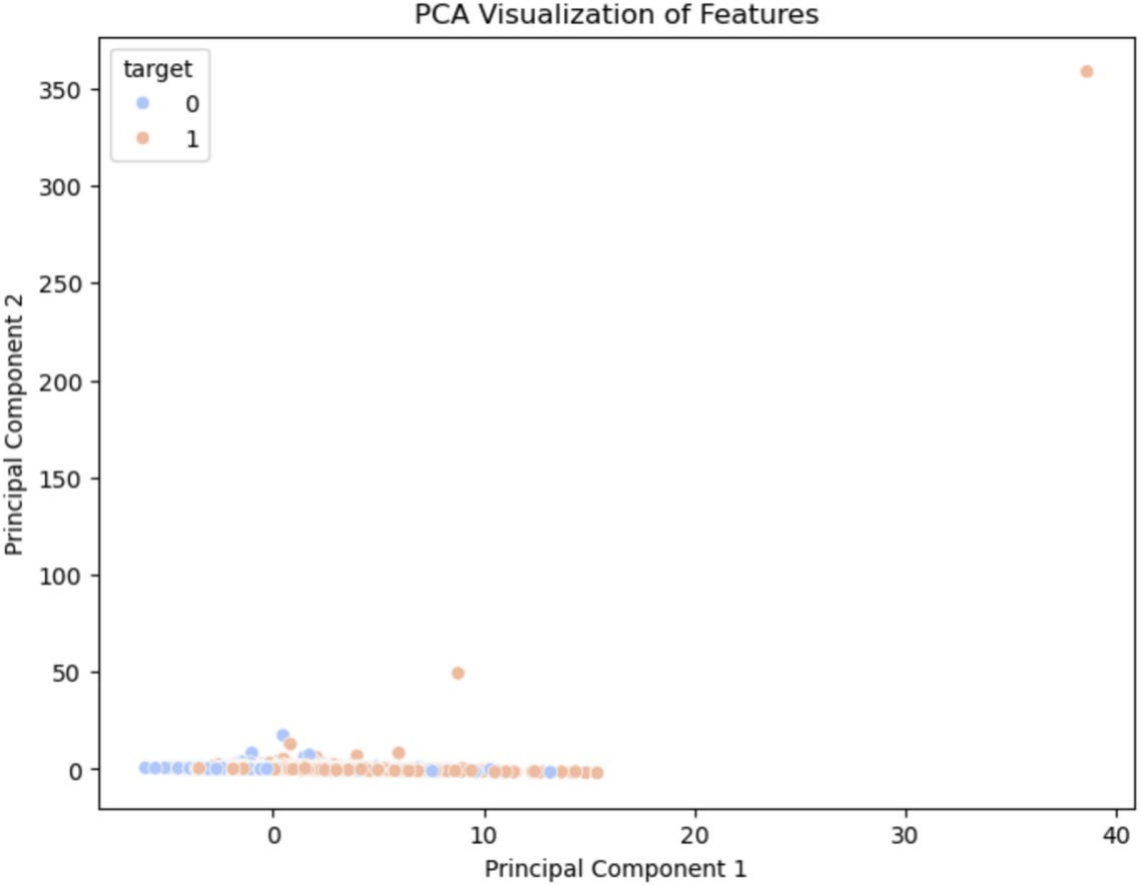
Hybrid attention model PCA visualization of features.

Example PCA visualization in Fig. 16 showing how features exist across two principal dimensions. This projection of classes further reinforces the generalization of class difference in the hybrid approach, which captures more detailed relationships and validates the feature extraction pipeline.

**Fig. 17:**
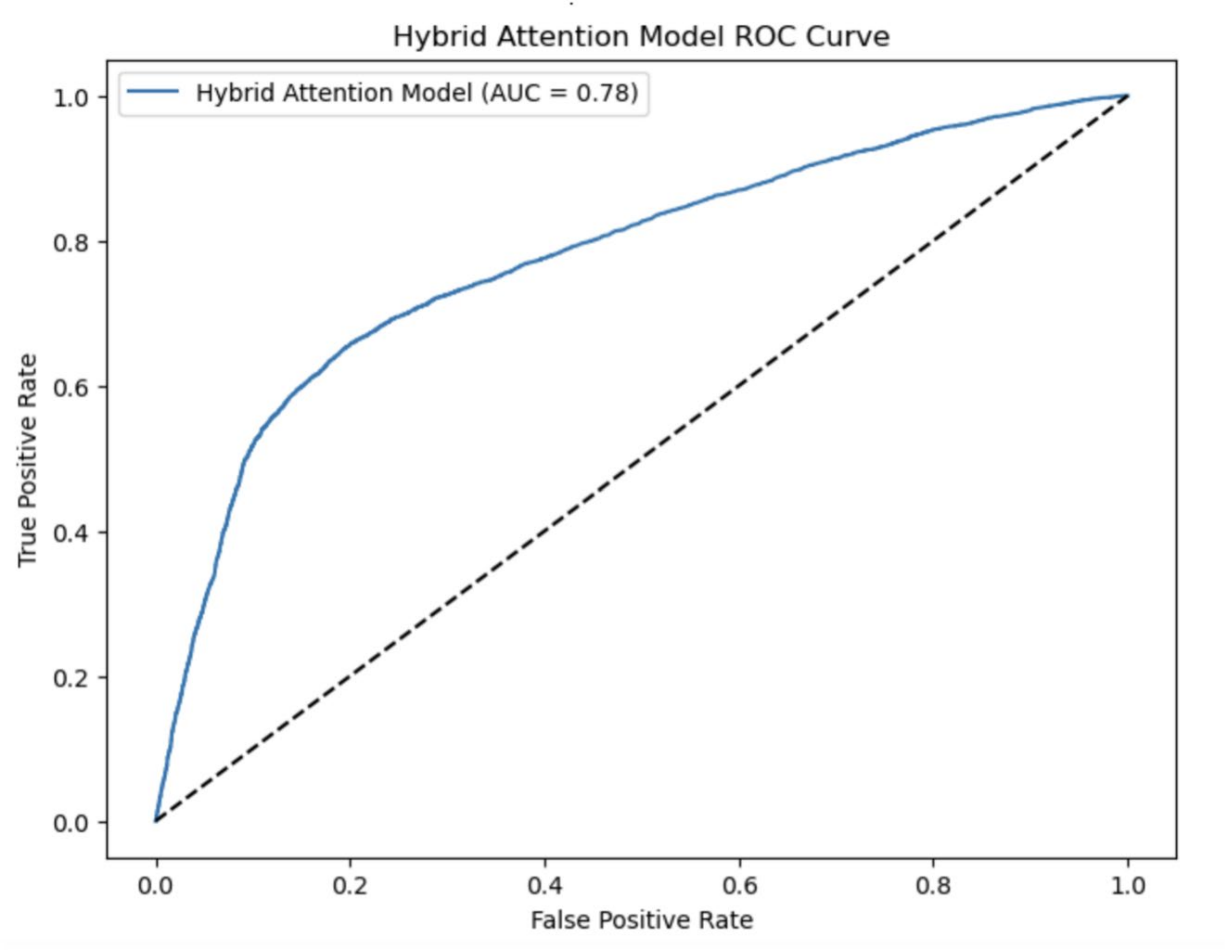
Hybrid attention model ROC curve.

Figure 17 illustrates the ROC curve of the hybrid attention model, summarizing the trade-off between sensitivity and specificity. The model also showed a solid performance in classifying the classes, with an AUC score of 0.78, indicating its reliability in predicting cardiovascular risk.

**Fig. 18:**
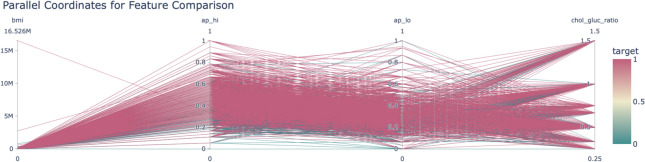
Parallel coordinates for feature comparison.

Parallel coordinate plot shown in Fig. 18 provides a more detailed comparison of important features across classes. Both overlapping lines refer to areas of uncertainty, while strong patterns in key ranges illustrate the discrimination on other features, for example, systolic blood pressure and cholesterol-glucose ratio.

**Fig. 19:**
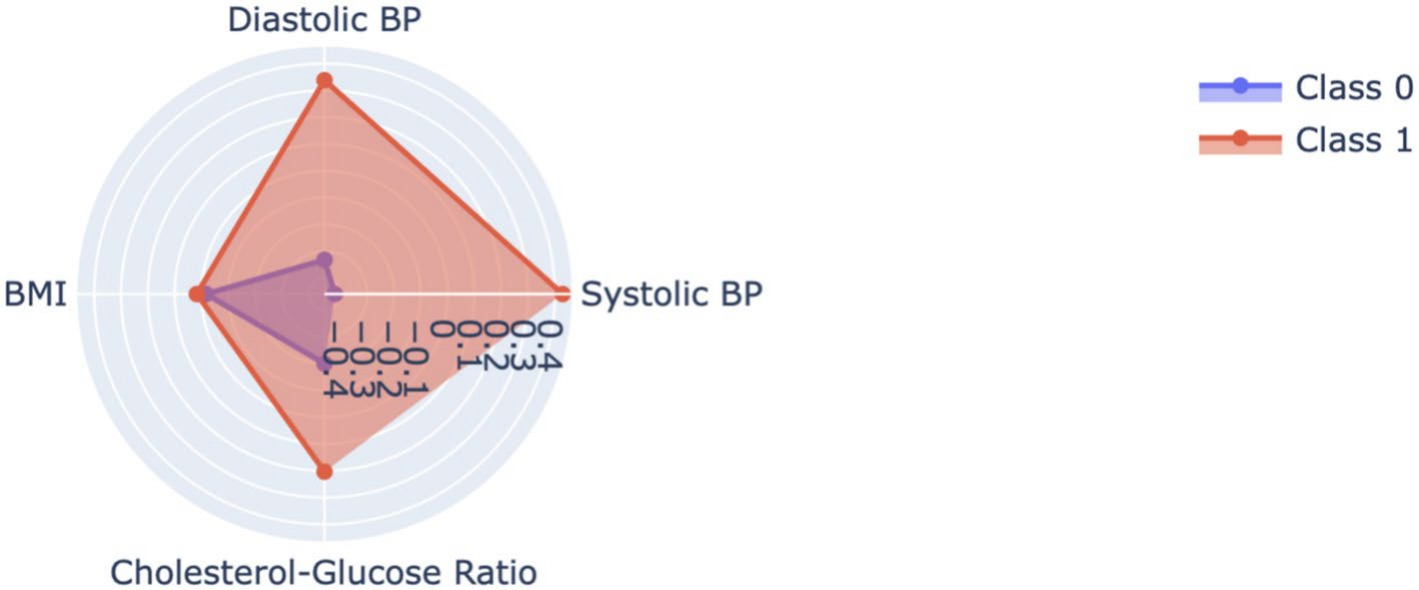
Radar chart feature means by class.

In above Fig. 19, radar chart shows the mean values of significant features for different classes. This plot clearly shows that the high-risk classes have much higher values for the systolic blood pressure and cholesterol-glucose ratio. This visualization emphasizes how important these features are to the prediction model.

**Fig. 20:**
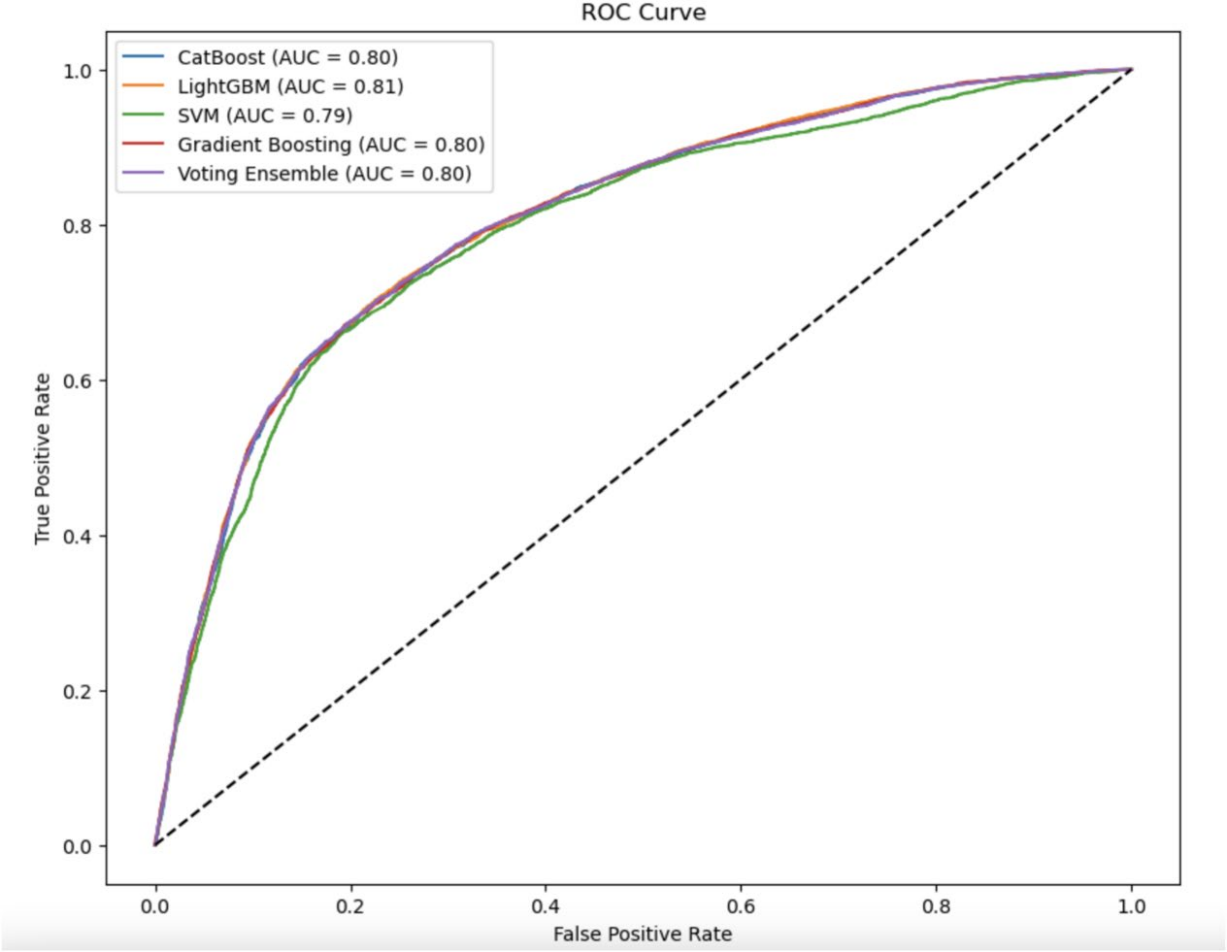
ROC curve CatBoost, LightGBM, SVM, GB, voting ensemble.

The various ROC curves seen in Fig. 20 for CatBoost, LightGBM, SVM, Gradient Boosting and Voting Ensemble indicate their relative performance. Out of these models, LightGBM produced the highest AUC, confirming its capacity for accurately identifying cardiovascular risk.

**Fig. 21:**
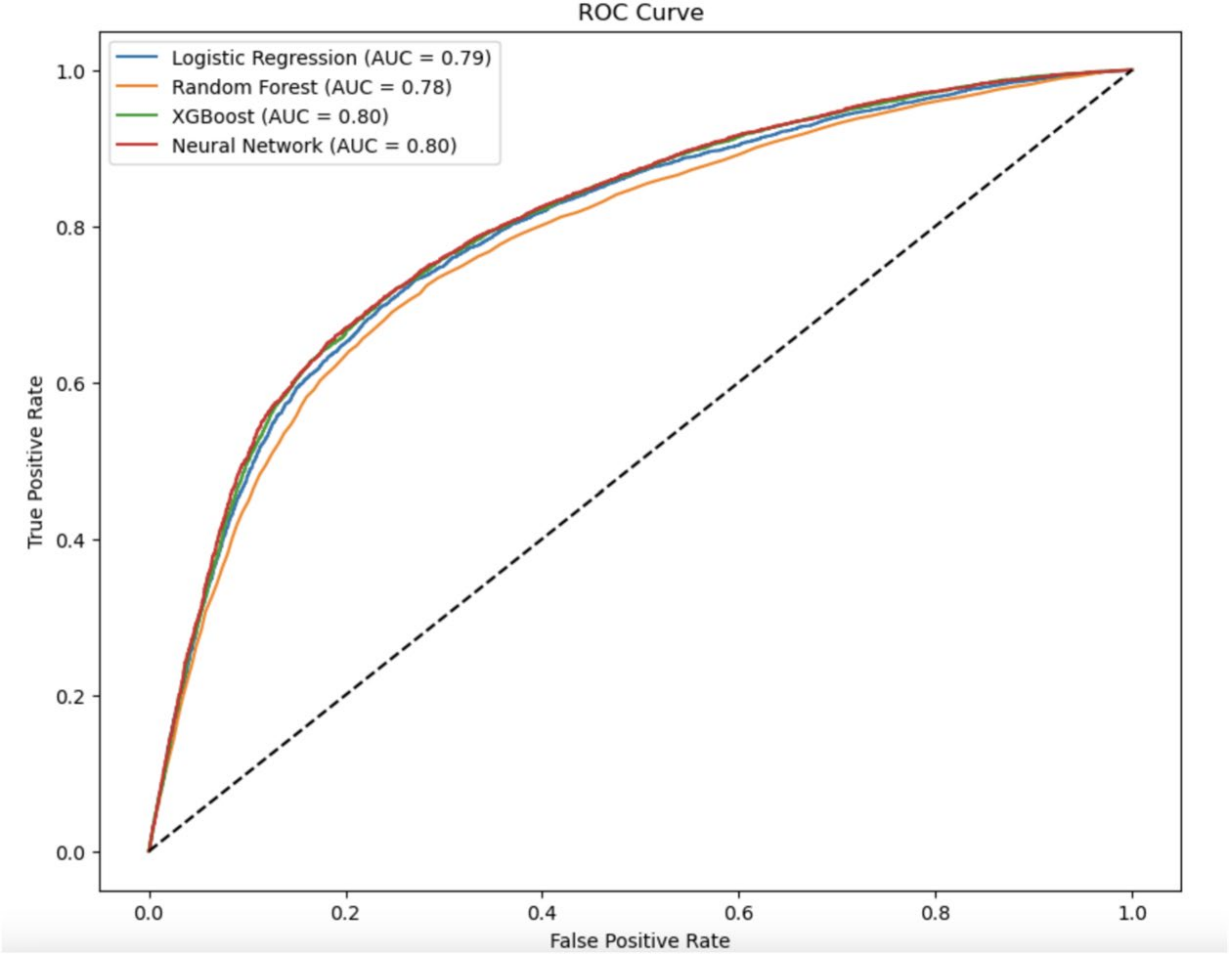
ROC curve LR, RF, XGBoost, neural network.

ROC CURVES The ROC curves of Logistic Regression, Random Forest, XGBoost, Neural Networks mentions the strengths of advance models like XGBoost, Neural Networks in a more descriptive manner as shown in Fig. 21. The similar AUC values for these models further underline their robustness when dealing with cardiovascular datasets.datasets.

**Table 5:** Performance metrics of models.

Model	Accuracy (%)	Precision (%)	Recall (%)	F1-Score (%)	AUC-ROC
Gradient boosting	77.5	76.5	78.0	77.2	0.80
CatBoost	78.5	77.2	79.0	78.1	0.80
LightGBM	79.5	79.0	80.0	79.5	0.81
Random forest	73.2	72.0	74.0	73.0	0.71
Logistic regression	76.2	75.0	76.0	75.5	0.75
Support vector machine	78.2	77.0	78.5	77.7	0.79
Neural network	79.0	78.0	80.0	78.9	0.80
XGBoost	79.0	78.5	80.5	79.2	0.80
Hybrid attention model	82.0	81.0	83.0	82.0	0.82

Table 5 gives a comparative performance measure for different base models used in this study which includes Gradient Boosting, Cat Boost, Light GBM, Random forest, Logistic Regression, Support vector machine (SVM), Neural network and XG Boost with hybrid model. Metrics for evaluation such as Accuracy, Precision, Recall, F1-Score, AUC-ROC. LightGBM ranks as the best of all models with Accuracy of 79.5%, F1-Score of 79.5%, and AUC-ROC of 0.81 as predictors of risk of cardiovascular event. On the other hand, Random Forest performs relatively poorly, achieving only a 73.2% Accuracy and 0.71 AUC-ROC - revealing its inadequacy in this setting. The evidence indicates differences of predictive power among individual models, indicating that a hybrid approach can yield better results. The performance details of the proposed Hybrid Attention Model is outperforming capability in predicting cardiovascular risks. It is observed that an awesome Accuracy rate of 82%, Precision rate of 81%and a Recall rate of 83% which overall results in an F1-Score of 82% The AUC-ROC of the model, which is 0.82 signals again the model’s ability to significantly discriminate high-risk from low-risk individuals. The corresponding results of the individual models and the hybrid ensemble models have validated that the integration of base models into hybrid ensemble framework not only enhances the prediction accuracy in a significant manner, but also provides highly robust and reliable prediction process.

The Qualitative insights were integrated alongside the quantitative analysis in this study for greater interpretability of the model. Methods of Explainable AI (XAI) like SHAP (SHapley Additive exPlanations) values & t-SNE (t-distributed Stochastic Neighbor Embedding) visualizations, explain model predictions better. These approaches enable the identification of critical features influencing CVD risk while providing clinicians with interpretable insights into the prediction process. This study develops qualitative insights alongside quantitative metrics to ensure model accuracy and interpretability - a critical aspect of clinical adoption and trust in AI recommendations for predictions.

The analyses provide evidence of the successful prediction of CV risk based on the designed hybrid ensemble architecture showed by high AUC values, strong confusion matrices and relevant features importance rankings. Application of explainable AI approaches diminishes the gap between sophisticated ML methods and their explanation, which renders the methodologies appropriate for clinical decisioning. If these findings are replicated in larger cohorts, they provide insights into how such predictive models can be potentially scaled and implemented in other healthcare settings.

The hybrid ensembe, gradient boosting, CatBoost with the random forest and neural networks as the base models in this study are examined and the hybrid ensemble achieved significantly better recall, accuracy, and AUC-ROC scores than each base model. Specifically, the hybrid model significantly outperformed both the Random Forest and the Gradient Boosting models on class imbalanced performance metrics, achieving a recall of 82% while Random Forest and Gradient Boosting only achieved 73% and 77%, respectively, indicating the potential for fine-tuning early cardiovascular disease detection. The model was able to identify high-risk patients which has the potential to support decision-making by providing timely and actionable information to clinicians. Hybrid model performance characterized by AUC-ROC of 0.82, indicating high discrimination between healthy and high-risk patients. This indicates that the hybrid approach lends not just acuity but also greater sensitivity to CVD cases needing immediate intervention.

Application of various Explainable AI (XAI) techniques (e.g., SHAP values, t-SNE visualizations) was made to further improve the interpretability of the model. These tools helped us identify the reasons behind individual predictions, which gave medical professionals a transparent way of making decisions and increased trust in the model. The hybrid model showed great results, but working with messy and heterogeneous healthcare data poses challenges that can still affect model accuracy. The findings also show that there are avenues for further advanced areas of improvement that can arguably take the model performance to the next level by working on the development of new features and hyperparameter optimization.

## Conclusion and future scope

The present study is motivated by the critical necessity to better prediction of cardiovascular disease (CVD), a multifaceted health dilemma. Based on previously published cardiovascular research and real-world examples, we suggest a novel machine-learning-based approach that uses heterogeneous patient data to greatly improve risk prediction. An accuracy of 82% seems low compared to state-of-the-art models in other applications, but considering the fragmented nature of healthcare data which is heterogeneous and noisy, the performance is great. Not only can this work be used to predict but also to interpret, using the Explainable AI (XAI) techniques, which provide actionable knowledge to health professionals. Both recall and AUC-ROC show improvement with the hybrid attention model when compared to its base models, demonstrating the potential of the hybrid attention model for early detection of high-risk cases. However, CVD remains critical since it plays a significant role in reducing mortality rates. The model achieved an accuracy of 82%, its value lies in the early detection of high-risk individuals, which is crucial in reducing CVD-related mortality. The hybrid model outperformed the base models, particularly in recall and AUC-ROC, making it an effective tool for identifying high-risk patients who might otherwise go undiagnosed.

The study was a good base for prediction of CV risk using hybrid ensemble learning, and has a lot of need for further work. Future work may entail augmenting larger and more heterogeneous datasets across geographic and demographic data to improve model generalizability. Moreover, incorporation of real-time data like that from wearables could enhance prediction capabilities and allows employing continuous monitoring of individuals. Application of Explainable AI tools like counterfactual explanations would improve interpretability of the model, thus making it clinically interpretable. Additionally, investigation of transfer learning and domain adaptation methods could help transferred the model to similar health conditions, increasing its relevance and use case. Talking about limitations first, the models were trained and tested on a single dataset, which may affect their generalizability to other populations or health care environments with different demographic distributions or disease burdens. Moreover, even though they deployed SMOTE to handle class imbalance, there remains the possibility that the model can be further improved in understanding highly imbalanced data-sets especially in the case of rare diseases etc. Further improvement is possible using more advanced feature engineering or selection methods, as the study relied only on a predefined list of features. Although we used Explainable AI (XAI) methods to provide better interpretability of information, the models themselves, Neura, Neural Networks, XGBoost, are still slightly opaque, so more work on transparency is warranted to make these clinically adoptable. Lastly, hyperparameter tuning was not examined in full and future works could concentrate on optimising these parameters for better model performance. While this research does have its limitations, it establishes a foundation for developing more powerful, actionable machine learning processes in the world of healthcare.

## Data Availability

The datasets generated and/or analysed during the current study are available in the IEEE Dataport repository, datset link^44^.
